# Dual-Path Optimization Strategy for High-Efficiency Chalcogenide Perovskite Solar Cells: Bulk Bandgap Engineering and Advanced Interfacial Design

**DOI:** 10.1021/acsomega.6c00626

**Published:** 2026-05-05

**Authors:** Hichem Bencherif, Mohamed Amir Abdi, Ziyad Younsi, Tarak Hidouri, Francesco Gussepe Della Corte

**Affiliations:** † LEREESI Laboratory, HNS-RE2SD, 05078 Batna, Algeria; ‡ Department of Mathematical, Physical and Computer Sciences, University of Parma, 43124 Parma, Italy; § Department of Electrical Engineering and Information Technologies (DIETI), 9307 Università Degli Studi di Napoli Federico II, 80125 Napoli, Italy

## Abstract

Chalcogenide perovskites offer a stable, high-performance alternative to hybrid perovskites. However, the full optoelectronic potential associated with their tunable bandgap remains underutilized. This study introduces a dual-path optimization strategy in lead-free BaZr_1-x_Ti_x_S_3_ chalcogenide perovskite that synergistically combines the implementation of a compositionally graded bandgap in BaZr_1-x_Ti_x_S_3_ using a β-function profile with advanced interfacial design and the insertion of WS_2_ electron transport layer (ETL). Precise control of the Zr/Ti compositional gradient within the absorber, governed by a β-function profile (*x*: 0–0.6), establishes a continuous internal energy field that enhances broadband photon harvesting and promotes efficient charge separation. Concurrently, the integration of WS_2_ in FTO/WS_2_/BaZr_1-x_Ti_x_S_3_/Cu_2_O/Au suppresses interfacial recombination and facilitates efficient charge extraction, thereby reducing charge loss. Solar Cell Capacitance Simulator – One Dimension (SCAPS-1D) simulations under AM1.5G illumination indicate that this co-optimized architecture can achieve a power conversion efficiency (PCE) of 24.22%. These results demonstrate that coordinated optimization of the absorber’s electronic landscape and the charge-selective contact is critical for unlocking the high-efficiency potential of emerging perovskite-inspired materials, providing a holistic design framework for next-generation photovoltaics.

## Graphical abstract

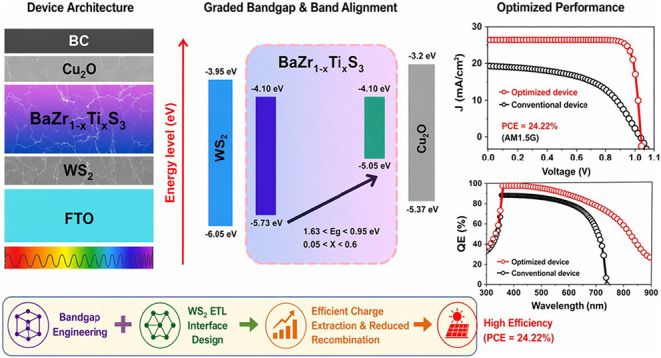

## 1 Introduction

Perovskite solar cells (PSCs), which combine high efficiency, cheap manufacturing costs, and customizable optoelectronic features, have become a game-changing breakthrough in the field of photovoltaics the past decade,.^1–5^ Perovskites, particularly organic–inorganic halide perovskites, have demonstrated remarkable PCEs exceeding 27% in laboratory settings, highlighting their rapid progress and strong potential for next-generation photovoltaic and optoelectronic devices.^6,7^ In addition to high efficiency, these materials exhibit tunable bandgaps, engineerable interfaces, and solution-processable fabrication, enabling straightforward integration into tandem and multijunction solar modules.^7^ Their combination of excellent optoelectronic properties, stability, and versatile processing routes positions halide perovskites as highly promising candidates for both efficient solar energy harvesting and advanced optoelectronic applications.^7^ However, problems including instability in environmental conditions and the usage of hazardous lead in their composition make them less economically appealing.^8^ To address these challenges, researchers have explored alternative perovskite materials, including chalcogenide perovskites, which exhibit superior stability and nontoxicity.^9^ Due to its exceptional optoelectronic characteristics, such as a good bandgap, significant absorption coefficient, and chemical stability, barium zirconium sulfide (BaZrS_3_) has established itself as a potential contender among these.^9^ BaZrS_3_ crystallizes in a GdFeO_3_-type structure and exhibits a direct bandgap of approximately 1.60–1.80 eV, with an absorption coefficient exceeding 10^5^ cm^–1^, outperforming many conventional absorption materials (10^4^ cm^–1^).^10,11^ BaZrS_3_ stands out as a leading candidate for photovoltaics due to its unique advantages: (i) it is lead-free and relatively simple to synthesize; (ii) it demonstrates outstanding carrier mobility; (iii) it is composed of economical, Earth-abundant, and nontoxic elements; and (iv) its absorption spectrum aligns closely with the maximum solar radiation wavelength.^12^ This material has also proven to be highly stable, enduring temperatures up to 600 °C, and even submersion in water.^13–15^ One of the most remarkable features of chalcogenide perovskites such as BaZrS_3_ is their tunable bandgap, which enables precise control over optical absorption and spectral response. This tunability allows for effective engineering of solar spectrum utilization, thereby improving the potential efficiency of photovoltaic devices within the limits of the Shockley–Queisser framework.^16,17^

Interface engineering in B-site alloyed perovskite solar cells has emerged as a key strategy for performance enhancement. By tuning the B-site composition, it is possible to simultaneously modify the electronic band structure, defect formation energetics, and interfacial band alignment, thereby reducing nonradiative recombination losses and improving carrier extraction. These effects collectively contribute to improved device performance by optimizing both bulk and interfacial electronic properties. Despite these advantages, BaZrS_3_-based solar cells still face efficiency limitations, primarily due to incomplete charge carrier extraction and recombination losses, particularly at interfaces. This highlights the importance of advanced interface and band engineering strategies to further improve device performance.

To address these limitations, this study presents a novel simulation-based approach that combines two key strategies: (i) the implementation of a compositionally graded bandgap in BaZr_1–*x*_Ti_*x*_S_3_ using a β-function profile, and (ii) the integration of WS_2_ as an ETL. While BaZrS_3_ and its alloyed derivatives have shown promise as stable, lead-free absorbers. Previous studies have not systematically explored the synergy between bandgap grading and ETL optimization in this material system. Our work demonstrates, through detailed SCAPS-1D simulations, how this proposed design can significantly enhance device performance, achieving a PCE of 24.22%. This study contributes to the development of next-generation renewable energy technologies by highlighting the significance of bandgap engineering and improved ETLs in creating stable, high-efficiency, and eco-friendly perovskite solar cells.

## 2 Modeling Framework

This study focuses on the simulation and optimization of a PSC with an n-i-p vertical configuration that comprises multiple functional layers. The structure incorporates a WS_2_ layer serving as ETL, and a BaZr_1–*x*_Ti_*x*_S_3_ layer functioning as the light-absorbing layer. The Cu_2_O layer is used as a hole transporting layer (HTL) while Gold (Au) and Fluorine-doped Tin Oxide (FTO) are selected as the back and front contact, respectively. Figure 1 presents the complete device architecture, illustrating the spatial arrangement of the layers and the respective materials used.

**Figure 1:**
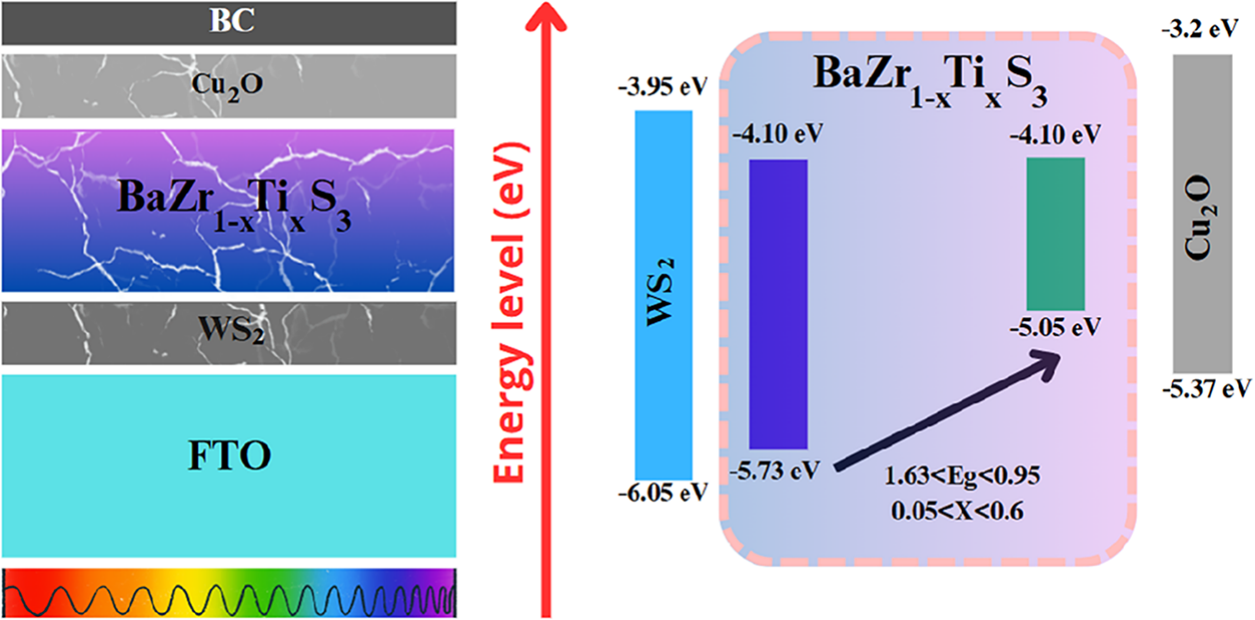
Cross-sectional view of the investigated solar cell and its corresponding band diagram.

To evaluate the performance of the suggested PSC, the SCAPS-1D (Solar Cell Capacitance Simulator) was employed.^18^ Material characteristics including bandgap, mobility, and electron affinity, for every layer were among the simulation inputs that were specified using values from the literature. Details regarding bulk and interface defects are also integrated into the modeling framework to enhance accuracy. Detailed information on all parameters used in the simulation is provided in Tables 1, 2, and 3. Our prior research has confirmed the accuracy of the developed simulation framework, showing a significant agreement with experimental results and proving the dependability of our modeling technique.^19–22^

**Table 1:** Design Parameters

parameters	FTO^23^	WS_2_ ^24^	BaZr_0.95_Ti_0.05_S_3_ ^25^	Cu_2_O^26^
Thickness (nm)	500	60	500	100
*E*_g_ (eV)	3.50	2.1	1.63	2.17
χ (eV)	4.00	3.95	4.10	3.20
Permittivity	9.00	13.6	9.60	7.11
*N*_c_	2.2 × 10^18^	2 × 10^18^	2.2 × 10^18^	2.02 × 10^17^
*N*_v_	1.8 × 10^19^	2 × 10^19^	1.8 × 10^19^	1.1 × 10^17^
Thermal velocity for electrons/holes	1 × 10^7^	1 × 10^7^	1 × 10^7^	1 × 10^7^
Electron mobility	20	250	0.017	200
Hole mobility	10	100	0.059	80
*N*_D_	1 × 10^18^	1 × 10^19^	1 × 10^12^	
*N*_A_			1 × 10^12^	1 × 10^18^
Defects concentration *N*_t_	1 × 10^15^	1 × 10^14^	1 × 10^15^	1 × 10^15^

**Table 2:** Defects Specifications (Data Collected from Refs 26,27 )

parameters	FTO	WS_2_	BaZr_0.95_Ti_0.05_S_3_	Cu_2_O
Defect type	Neutral	Neutral	Neutral	Neutral
Capture cross-section (cm^2^)	1.0 × 10^–15^	1.0 × 10^–15^	1.0 × 10^–15^	1.0 × 10^–15^
*E*_t_ (eV)	>Ev	>Ev	>Ev	>Ev
Energy level w.r.t reference (eV)	0.6	0.6	0.6	0.6
Characteristics energy	0.1	0.1	0.1	0.1
Defects concentration *N*_t_ (cm^–3^)	10^15^	10^14^–10^15^	10^15^	10^15^

**Table 3:** Interface Defects Details (Data Collected from Refs 26,27 )

parameters	WS_2_/BaZr_0.95_Ti_0.05_S_3_	BaZr_0.95_Ti_0.05_S_3_/ Cu_2_O
Defect type	Neutral	Neutral
Characteristics energy	0.6	0.05
*E*_t_	Single	Single
Energy distribution	1.0 × 10^–15^	1.0 × 10^–15^
Defects concentration *N*_t_	1.0 × 10^11^	1.0 × 10^12^

The purpose of the simulation was to identify key performance indicators such as *J*_SC_, *V*_OC_, FF, and overall PCE. The analysis focused on understanding how graded bandgap profiles influence the device’s performance. This computational method enables design optimization techniques to increase the efficiency of PSCs and provides insightful information about their functional characteristics.

Standard test settings, such as an AM 1.5G light spectrum at 300 K, were used for all simulations.

The relatively low carrier mobility values used for the BaZr_0.95_Ti_0.05_S_3_ layer, adopted from BaZrS3 SCAPS-1D study,^29^ are consistent with chalcogenide perovskites where transport is limited by defect- and phonon-induced scattering. In this framework, mobility is intrinsically related to carrier lifetime and defect density through scattering mechanisms (Matthiessen’s rule) and thus reflects nonideal material quality. Therefore, these conservative parameters may lead to an underestimation of absolute performance, while preserving the validity of the observed trends. This section of the study focuses on engineering the bandgap of the absorber layer in BaZr_1–*x*_Ti_*x*_S_3_. To improve photon absorption, the bandgap is tuned by changing the content throughout the entire thickness of BaZr_1–*x*_Ti_*x*_S_3_. A β-function grading profile is utilized to achieve the desired compositional variation.

## 3 Experimental Feasibility

The fabrication of a compositional gradient in BaZr_1–*x*_Ti_*x*_S_3_ thin-film absorbers presents significant experimental challenges, primarily in achieving precise spatial control over Ti concentration throughout the film thickness. However, recent advances in thin-film deposition technologies offer promising approaches to realize such graded profiles. Techniques such as combinatorial sputtering, where multiple targets with different compositions are cosputtered with controlled power gradients, can enable smooth compositional variation across the substrate.^30^ Pulsed laser deposition with sequential or simultaneous ablation of BaZrS_3_ and BaTiS_3_ targets also allows for fine-tuning of the local composition layer-by-layer.^31^ Chemical vapor deposition and atomic layer deposition provide additional avenues for atomic-level control of composition through precursor flow modulation.^32^ Recent studies have also demonstrated relatively simple strategies for fabricating bandgap-graded perovskite absorber layers through controlled compositional engineering during film formation. For example, graded perovskite films have been achieved using solution-processed compositional modulation approaches, confirming the practical feasibility of realizing graded band structures in photovoltaic absorbers.^33^ Although these methods require careful optimization to avoid phase segregation and maintain film quality, they have been successfully applied in other complex oxide and chalcogenide systems to produce bandgap-graded layers.^34,35^ These developments suggest that the bandgap grading strategies proposed in our simulations are experimentally achievable, paving the way for practical realization of high-performance BaZr_1–*x*_Ti_*x*_S_3_-based photovoltaic devices.

## 4 Results and Discussion

### 4.1 Conventional Design Performance

W. Meng explored the variation in bandgap energy (*E*_g_) by adjusting the composition of zirconium (Zr) and titanium (Ti) in the BaZr_1–*x*_Ti_*x*_S_3_ material.^29^ When Ti is absent (*x* = 0), the *E*_g_ is stated to be 1.72 eV. However, as Ti is introduced, the *Eg* decreases. At *x* = 1, where Ti fully substitutes Zr, the *E*_g_ narrows significantly to approximately 0.61 eV. The bandgap values for intermediate compositions (0 < *x* < 1) are illustrated in Figure 2a. The precise control over the Zr/Ti compositional gradient (*x*) within the BaZr_1–*x*_Ti_*x*_S_3_ absorber layer is critical, as it directly engineers a corresponding gradient in the material’s *E*_g_. This variation in *E*_g_ enables the absorber to efficiently harvest photons across a broad solar spectrum, with wider-gap regions (high Zr, low *x*) absorbing high-energy photons and narrower-gap regions (high Ti, high *x*) absorbing lower-energy photons. Concomitantly, the changing composition induces gradients in optical constants, such as the refractive index and extinction coefficient, which can be tailored to minimize front-surface reflection and manage light trapping within the cell. Electrically, this intentional bandgap gradient creates a built-in quasi-electric field that provides a thermodynamic driving force for charge carrier separation. This field actively assists in sweeping photogenerated electrons and holes toward their respective contacts, thereby suppressing bulk recombination, enhancing carrier collection, and ultimately optimizing the solar cell’s overall power conversion efficiency.

**Figure 2:**
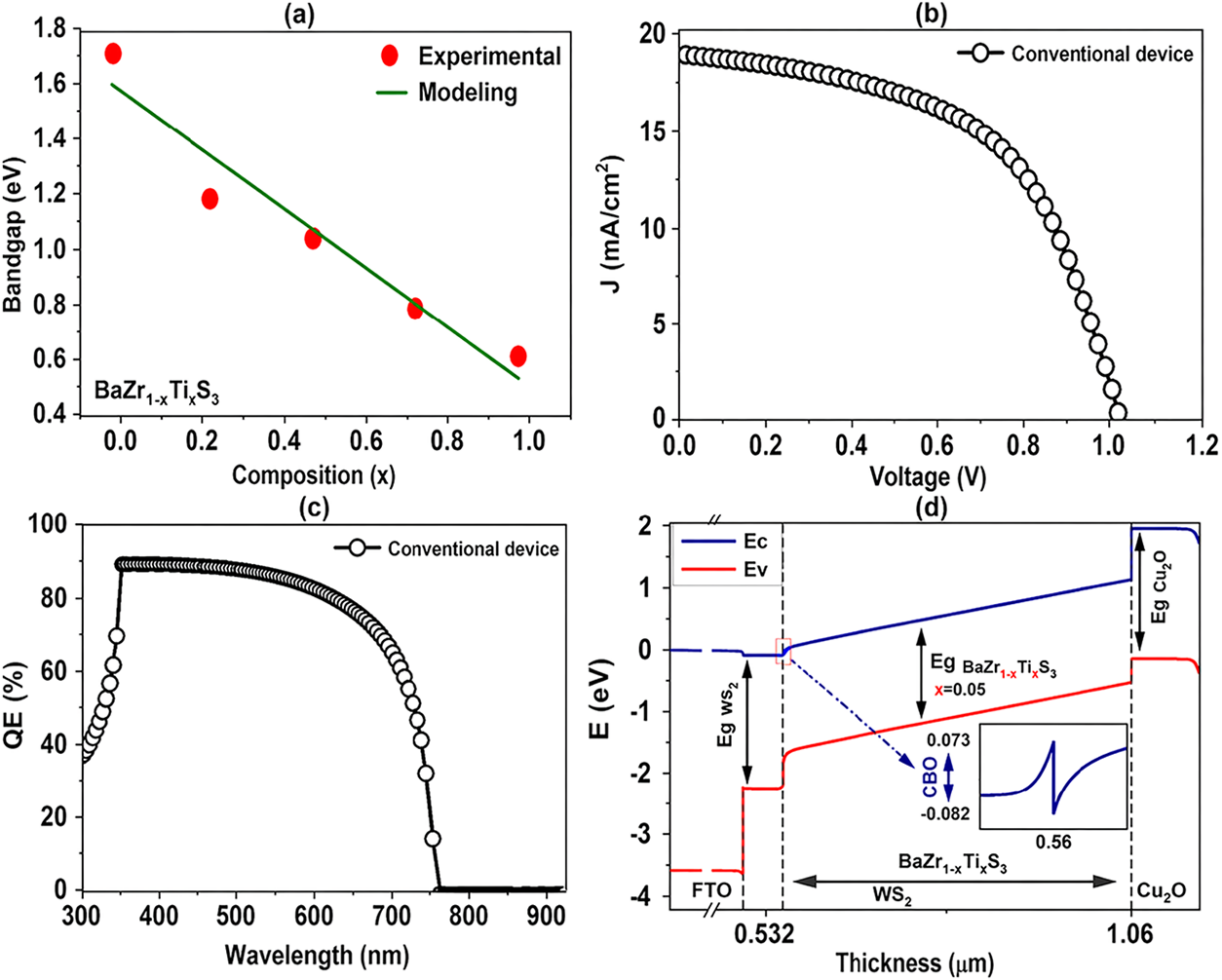
(a) Variation in bandgap of BaZr_1–*x*_Ti_*x*_S_3_ as a function of *x* content, obtained from numerical simulations (Reproduced from^28^). (b) *J–V* characteristic (c) QE, (d) Energy band diagram curves of n-i-p CPSC, obtained from numerical simulations.

Figure 2b,c shows the *J–V* curve and EQE for the standard BaZr_1–*x*_Ti_*x*_S_3_ PSCs utilizing WS_2_ as ETL material. As shown in Figure 2b, the device demonstrates a *J*_SC_ of 19.073 mA/cm^2^, a *V*_OC_ of 1.08 V, an FF of 52.92%, and an overall efficiency of 10.90%. These performance metrics are explained by the advantageous band alignment at the interface between WS_2_ and BaZr_1–*x*_Ti_*x*_S_3_, which lessens the effect of interface defects. Furthermore, Figure 2c shows that within the wavelength range of 300–760 nm, the device maintains strong performance. This is primarily due to the low surface recombination rate of WS_2_’s and its high diffusion length, which facilitate efficient charge transport and collection.

Figure 2d depicts the energy band diagram of the investigated PSC. The conduction band of WS_2_ is slightly higher than that of the BaZr_1–*x*_Ti_*x*_S_3_ absorber, resulting in a small spike-type conduction band offset (Δ*E* ≈ +0.15 eV). Such a small positive conduction band offset (CBO) is generally beneficial for photovoltaic devices, as it suppresses interfacial electron–hole recombination while still allowing efficient electron transport across the interface. In addition, a suitable valence band offset is formed at the BaZr_1–*x*_Ti_*x*_S_3_/Cu_2_O interface, which effectively blocks electron backflow and facilitates hole extraction toward the Cu_2_O hole transport layer. Therefore, the overall band alignment promotes efficient carrier separation and reduces recombination losses within the device.

### 4.2 Influence of β-Graded Bandgap

The β-function has four interpolation parameters, which are the left and right compositions (similar to the linear graded profile). In the case of BaZr_1–*x*_Ti_*x*_S_3_, it is found to be 0.05 on the left side (WS_2_/BaZr_1–*x*_Ti_*x*_S_3_) and between 0.05 and 1 on the right side (BaZr_1–*x*_Ti_*x*_S_3_/Cu_2_O interface). The two additional β-function parameters, denoted *a* and *b*, are initially set to unity, resulting in an approximately linear profile. This baseline configuration is used to optimize the bandgap at BaZr_1–*x*_Ti_*x*_S_3_/HTL interface. After optimizing *x* at BaZr_1–*x*_Ti_*x*_S_3_/Cu_2_O interface, we then proceed to optimize β-function parameters (*a* and *b*) to generate β-function profile configuration. The β-function is given as follows^36^

1 $$P_{A}+\left(P_{A}-P_{B}\right)\beta_{a,b}\left(\frac{y-y_{A}}{y_{B}-y_{A}}\right)$$

where *P_A_* and *P_B_* represent the values of the property at the start and end points of the grading region, respectively. The term $\left(\frac{y-y_{A}}{y_{B}-y_{A}}\right)$ normalizes the spatial position *y*, mapping it to a range between 0 and 1. The function β_*a,b*_ (*x*) is the regularized incomplete β-function, controlled by shape parameters a and b, which allows for a smooth and tunable transition between *P*_*A*_ and *P*_*B*_.

To determine the optimal Ti concentration at the bottom borders of the absorber layer (interface of BaZr_1–*x*_Ti_*x*_S_3_ /HTL), we varied the bottom edge composition, and the findings are illustrated in Figure 3. The collective fluctuation in Ti composition (at the BaZr_1–*x*_Ti_*x*_S_3_ /Cu_2_O interface) was investigated using PV parameters, *V*_OC_, *J*_SC_, FF, and PCE. It can be noticed that the *V*_OC_ is maximal when the Ti composition is maintained between 0.2 and 0.3 throughout the absorber layer, which corresponds to an *E*_g_ of around 1.5 eV. Figure 4 clearly shows how a tighter bandgap reduces the *V*_OC_. This is owing to higher reverse saturation current and increased recombination rates in materials with a narrower energy gap, which restrict the attainable voltage. Furthermore, a trend has been found for *J*_SC_, as increasing the Ti concentration from 0 to 1 has improved the *J*_SC_. Linearly graded BaZr_1–*x*_Ti_*x*_S_3_ PSC produced a maximum *J*_SC_ of 32 mA/cm^2^ at *x* = 1, corresponding to the minimum *E*_g_ of 1 eV. Lower *E*_g_ materials absorb more photons, resulting in higher carrier production and *J*_sc_. The FF has also showed enhancement by altering the Ti level from 0 to 0.5, after which it begins to deteriorate. PCE has demonstrated an important increase in linearly graded PSC by adjusting *x* from 0 to 0.6. The linear graded BaZr_1–*x*_Ti_*x*_S_3_ PSC delivers a maximum PCE of 14.9% at *x* = 0.55, which corresponds to a Zr-rich composition on top with an *E*_g_ of 1.63 eV and a Ti-rich composition on the bottom with an *E*_g_ of 1.28 eV.

**Figure 3:**
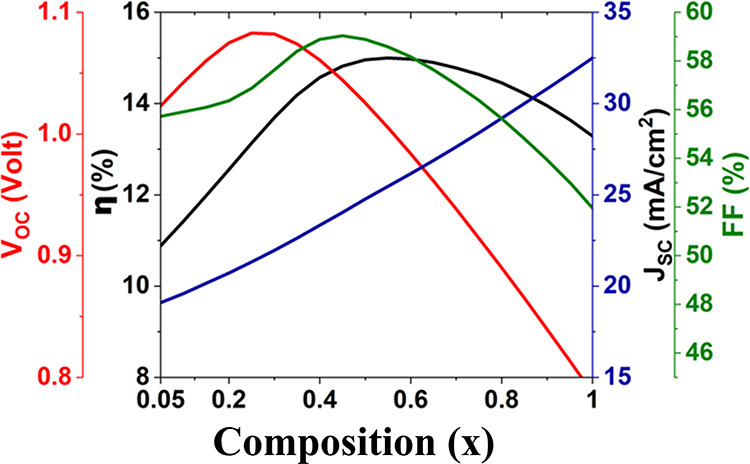
Profile of a linearly graded 500 nm-thick BaZr_1–*x*_Ti_*x*_S_3_ absorber layer in a PSC with WS_2_ as the ETL, obtained from numerical simulations.

**Figure 4:**
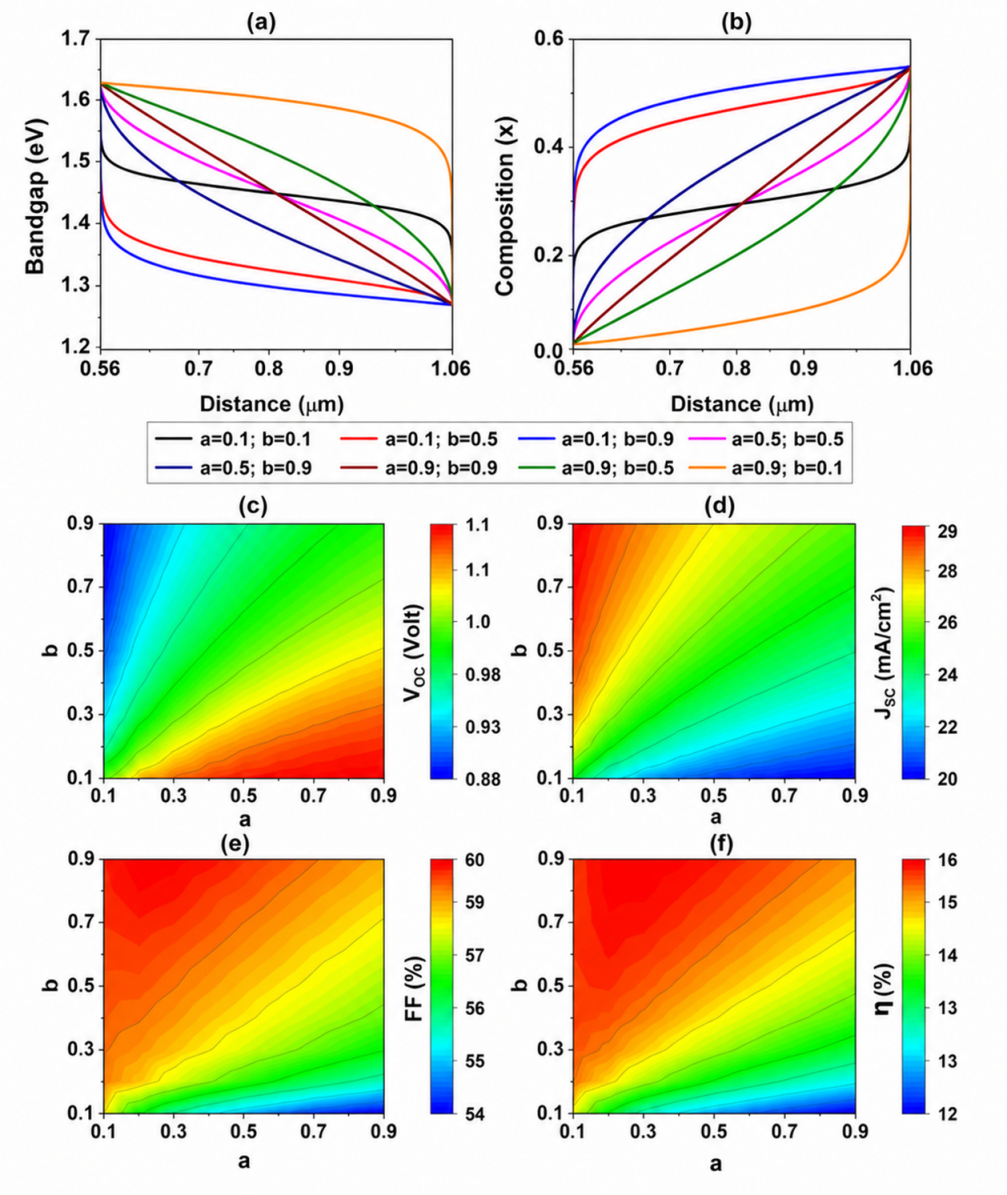
Profile of β-function-graded BaZr_1–*x*_Ti_*x*_S_3_ PSC: (a) variation of bandgap, (b) variation of composition *x*. Variation of “*a*” and “*b*” parameters of the β-profile of BaZr_1–*x*_Ti_*x*_S_3_ with WS_2_ as ETL, considering a left and right compositions of 0.05 and 0.55, respectively. Variation of (c) *V*_OC_, (d) *J*_SC_, (e) FF, and (f) efficiency, all obtained from numerical simulations.

Once the absorber has been linearly graded with the upper content set to *x* = 0.05 and the bottom composition set to *x* = 0.55, the PCE is 14.9% with *V*_OC_ = 1 V, *J*_SC_ = 25.45 mA/cm^2^, and FF = 58.58%. Further optimization was conducted by employing a β function grading profile at the bottom composition boundary, with parameters adjusted to align with a linear trend. This approach allowed controlled modulation of the compositional profile within the absorber’s bulk by tuning the *a* and *b* parameters of the β-function. These parameter adjustments influenced the energy bandgap across the bulk material, as shown in Figure 4. When *a* and *b* are both set to 1, the composition follows a linear variation. By varying *a* and *b*, a wide range of compositional gradings can be achieved, including linear, exponential, and more intricate polynomial profiles.

The contour plots presented in Figure 4(c–f) elucidate the relationships between the composition parameters *a* and *b* and their effects on key photovoltaic performance metrics: *V*_OC_, *J*_SC_, FF, and PCE of absorber layer with WS_2_ functioning as an ETL. The *J*_SC_ and *V*_OC_ metrics are significantly affected by variations in the profiles of *a* and *b*, which often exhibit a trade-off relationship. Specifically, the *V*_OC_ plot (Figure 4c) reveals a pronounced increase in open-circuit voltage as the values of composition *a* rise and *b* decrease, achieving a maximum *V*_OC_ of ∼1.1 V in the lower right region of the contour. In contrast, (Figure 4d) illustrates a consistent increase in *J*_*SC*_ with higher values of *b* and lower values of *a*, culminating in a peak *J*_SC_ of 29 mA/cm^2^. Furthermore, the FF plot (Figure 4e) and the PCE plot (Figure 4f) indicate significant enhancements in both FF and PCE at higher values of composition *b* and lower values of *a*. The FF increases from 54% to 60%, while the PCE reaches its maximum of 16% in the region where *a* ranges from 0.1 to 0.3 and *b* approaches 0.9. These results underscore the critical influence of compositional tuning on the photovoltaic performance of BaZr_1–*x*_Ti_*x*_S_3_ PSC with WS_2_ as an ETL.

### 4.3 Performance Comparison of Designs Using β Graded Bandgap

The *J–V* curve and QE comparison between the suggested design and a traditional device with a uniform bandgap are shown in Figure 5. As shown in Figure 5a, the proposed design with β-function graded profile exhibits superior performance compared to the conventional device, demonstrating enhanced *J*_SC_ (27.94 mA/cm^2^) and FF (59.538%), albeit with a slight decrease in *V*_OC_ (0.934 V). This performance improvement is related to the improved light absorption across a broader spectrum, which, in turn, enhances overall charge transport characteristics. However, the implementation of graded bandgap profile can also lead to increased carrier recombination, as evidenced by the observed reduction in *V*_OC_. The observed decrease in *V*_OC_ (1.03 V vs 1.08 V) in the optimized β-function graded design is mainly attributed to an increase in interfacial recombination associated with the stronger band bending introduced by the compositional grading. While this grading enhances carrier separation and transport, it can also slightly increase the electron–hole overlap near the interface, which raises recombination activity and consequently reduces *V*_OC_. Importantly, this trade-off is not fundamentally inherent to the β-function profile itself, but rather to the current implementation without additional interface passivation. Therefore, the *V*_OC_ loss can be mitigated through further interfacial engineering strategies, such as defect density reduction at the absorber/ETL interface, insertion of ultrathin passivation layers, or band offset tuning to reduce recombination velocity while preserving the favorable electric field distribution. From Figure 5b, it is obvious that EQEs of solar cells with a graded bandgap are higher than that of uniform bandgap cells owing to a boosted light absorption over a wider spectrum and an enhanced charge carrier extraction. The graded bandgap generates an internal electric field that reduces recombination losses and facilitates more efficient carrier collection, resulting in higher efficiency (15.54%).

**Figure 5:**
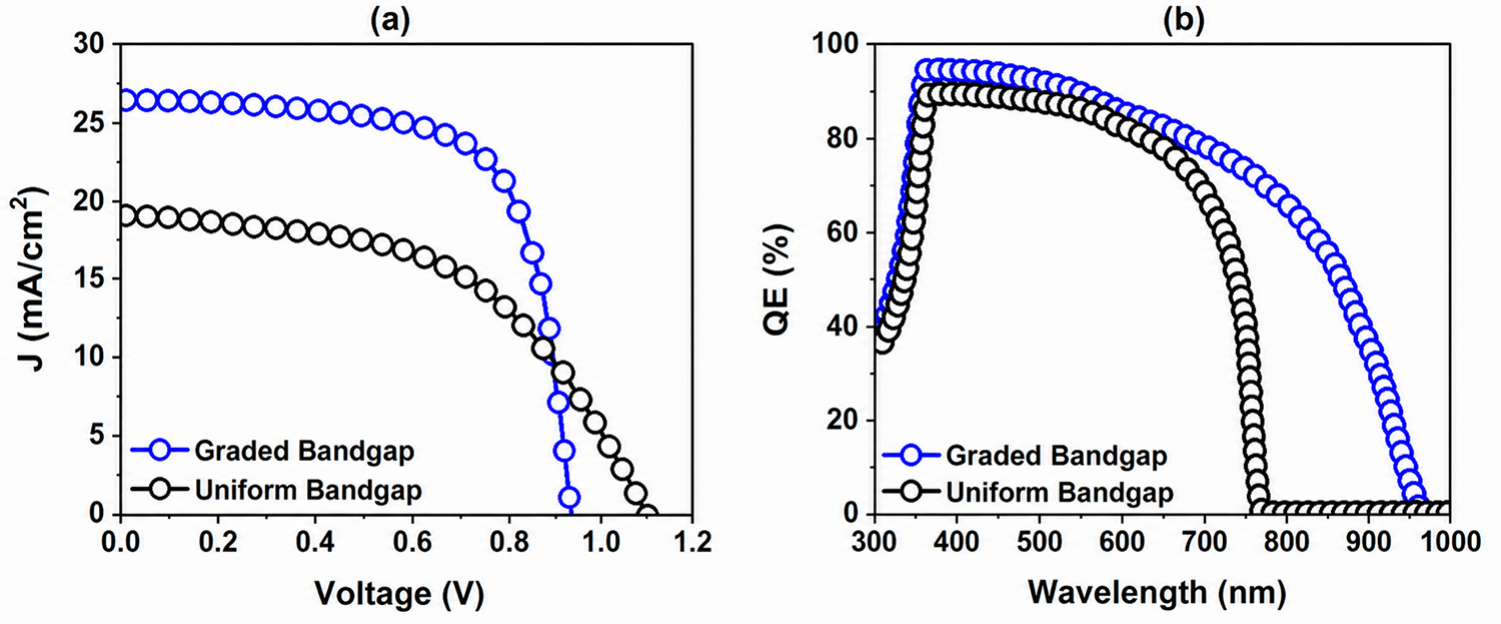
Comparison of (a) *J–V* characteristics and (b) EQE of the devices based on graded and uniform bandgap material, obtained from numerical simulations.

This graded bandgap profile induces a built-in internal electric field by gradually shifting the conduction and valence band edges. The resulting electric field promotes efficient charge carrier separation by driving electrons toward the ETL and holes toward the HTL. This directed carrier transport reduces the likelihood of electron–hole recombination within the absorber bulk, a common loss mechanism in uniform-bandgap devices where carrier extraction is primarily diffusion-driven. Consequently, the internal field generated by the β function grading enhances charge extraction efficiency and suppresses recombination losses, contributing significantly to the improved power conversion efficiency observed in the graded devices. This mechanism underscores the advantage of bandgap engineering strategies in optimizing perovskite solar cell performance.

The generation and recombination rates of the suggested and conventional designs are shown in Figure 6. The results indicate that device with a graded bandgap profile exhibits significantly higher generation rates compared to those with a uniform profile, attributed to the enhanced light absorption of the proposed design. Meanwhile, Figure 6b shows only a small difference in recombination rates between the two designs. These findings suggest that the graded bandgap design not only improves light absorption and carrier generation but also effectively controls recombination, leading to higher overall solar cell efficiency.

**Figure 6:**
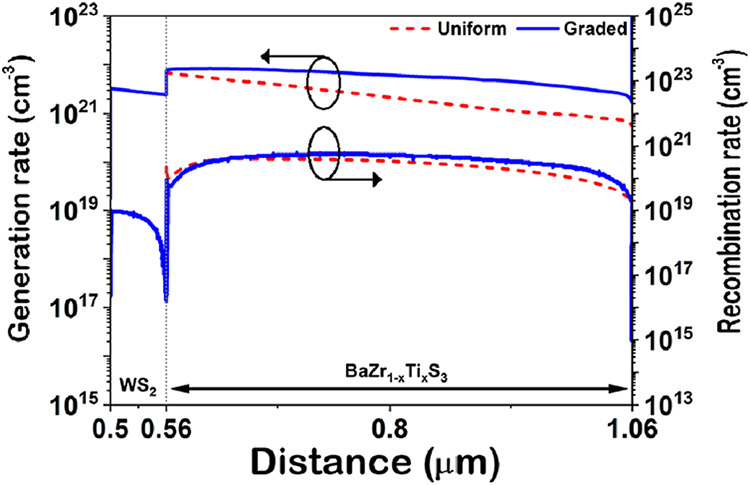
Generation and recombination rates of the baseline and the suggested designs, obtained from numerical simulations.

The band diagrams of the device under investigation, featuring uniform and β-function-graded absorber bandgaps, are presented in Figure 7, illustrating the impact of these profiles on device efficiency.

**Figure 7:**
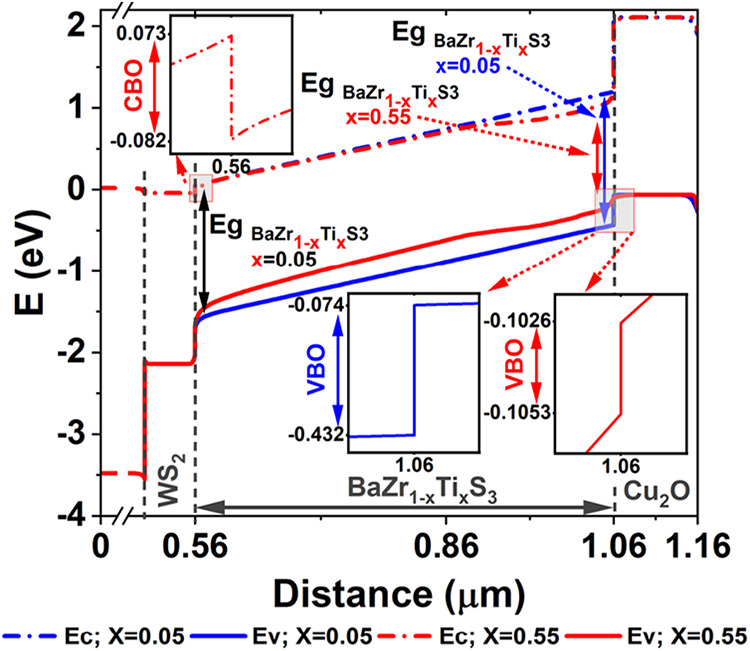
Energy band diagram comparison between the baseline and the suggested bandgap profile, obtained by numerical simulations. The β function is indicated in red, while the energy bands that correspond to uniform are displayed in blue.

According to the study, a “cliff-like” valence band offset (Δ*E*_v_ = −0.36 eV) is evident for the device with a uniform bandgap. At the BaZr_1–*x*_Ti_*x*_S_3_/Cu_2_O interface, this misalignment creates a barrier that hinders effective hole extraction, resulting in poor hole transport and increased recombination which reduces the device’s PCE. Conversely, for a β-function profile with *x* = 0.55, a matched band offset is established; however, hole transport into the HTL may still require overcoming an interfacial energy barrier.

### 4.4 Optimized Design

The effect of absorber layer thickness on the electrical characteristics of the suggested PSCs is shown in Figure 8a. The *V*_OC_ increases only slightly with thickness due to enhanced photon absorption. In contrast, the FF gradually decreases as thicker layers lead to increased recombination and higher series resistance. As the absorber thickness increases, the *J*_SC_ initially rises owing to improved light absorption. However, beyond a certain thickness, recombination losses become more significant, as photogenerated carriers must undergo longer transport distances to reach the contacts. Furthermore, the carrier collection efficiency decreases, resulting in a reduction in *J*_SC_. Indeed, there is a suitable thickness for an absorber where the balance between light absorption and carrier recombination is perfectly balanced. This study finds that the most effective thickness is between 200 and 300 nm, resulting in optimal performance for the device with a PCE around 17%.

**Figure 8:**
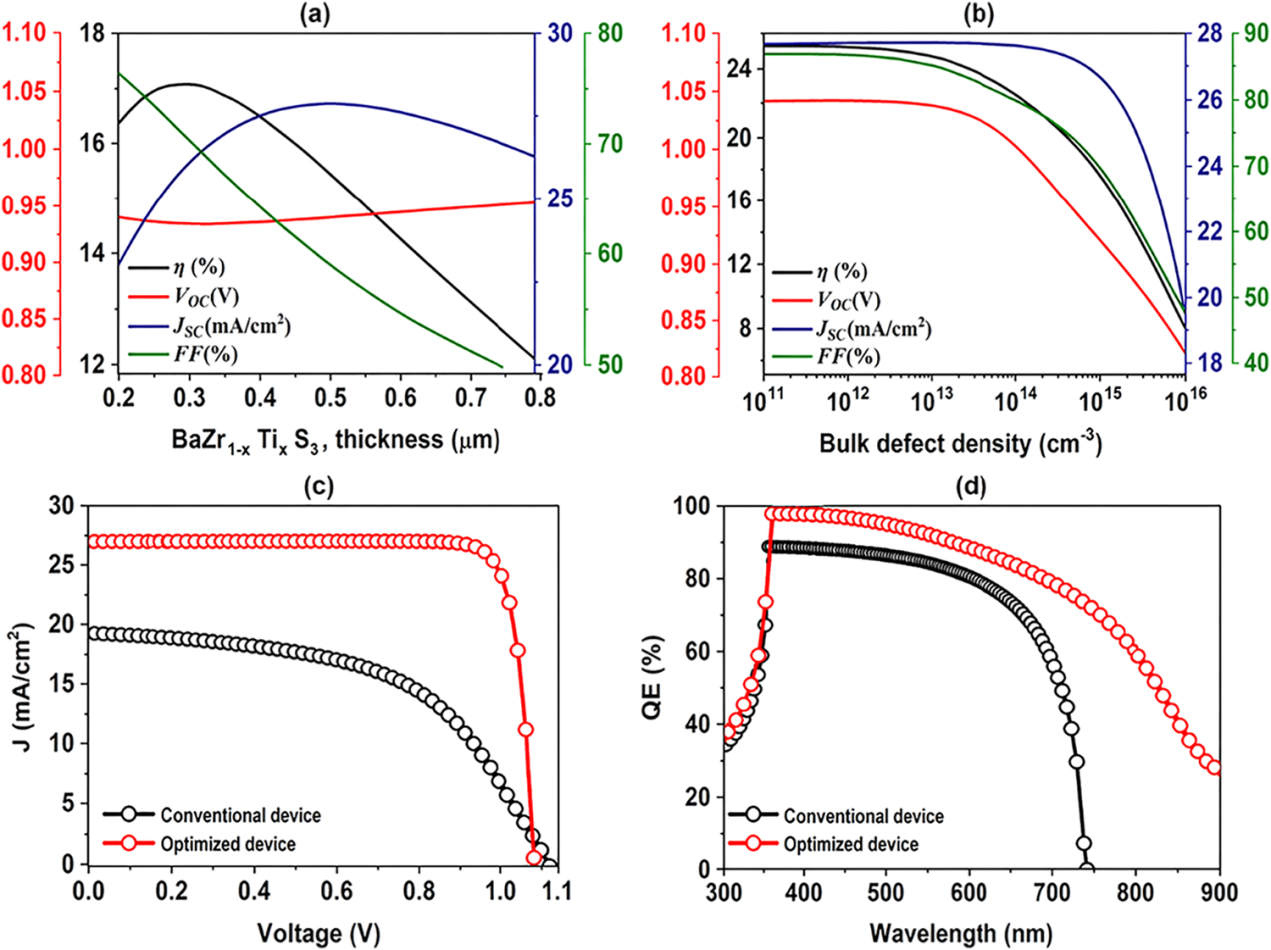
(a) Variation in absorber thickness. (b) Variation in bulk defect density in BaZr_1–*x*_Ti_*x*_S_3_ at a thickness of 300 nm. Comparison between the optimized and the baseline PSCs in terms of (c) *J–V* (d) EQE curves, obtained from numerical simulations.

In addition to bandgap tuning, the incorporation of Ti into BaZr_1–*x*_Ti_*x*_S_3_ may influence defect formation energetics. Substituting Zr^4+^ with Ti^4+^ modifies the local lattice environment and bonding characteristics, which can affect the stability and energy levels of intrinsic defects such as vacancies (e.g., *V*_S_, *V*_Zr_, *V*_Ti_), interstitials, and antisite defects. These changes can significantly impact carrier lifetimes and recombination dynamics. Although a detailed analysis of defect energetics across varying compositions would require first-principles calculations, we accounted for the influence of bulk defects on device performance by incorporating a representative trap state into the BaZr_1–*x*_Ti_*x*_S_3_ absorber in SCAPS-1D simulations. This defect was modeled as a neutral, single-level trap located at an energy *E*_t_ above the valence band maximum (*E*_v_), with a characteristic energy of 0.1 eV. The capture cross sections for both electrons and holes were set to 1 × 10^–15^ cm^2^, and the defect concentration *N*_t_ was assumed to be 1 × 10^15^ cm^–3^, consistent with reported values for chalcogenide perovskites. While this simplified model does not capture composition-dependent changes in defect formation energy or density, it allows us to assess the general impact of midgap recombination and trap-assisted losses under the influence of bandgap grading and interface engineering.

The study investigated how absorber defects impact the device outputs by varying absorber defect densities (*N*_t_) from 10^11^ cm^–3^ to 10^16^ cm^–3^. Figure 8b shows that the performance measures, including *V*_OC_, *J*_SC_, FF, and PCE, were steady but then decreased as fault density rises. The increase in defect density brings more defect states, traps, and recombination pathways into the material, reducing the overall performance.^29^ Although creating a material with an exceptionally low defect density may be difficult or impossible, optimizing manufacturing procedures and using appropriate components in PSCs might assist minimize defects while retaining other critical device features.

The *J–V* curves and EQE comparison between the optimized and the traditional design are shown in Figure 8c,d. The optimized design performs better than the traditional one in terms of *J*_SC_ and FF, as shown in Figure 8c. By establishing a Spike-like setup and utilizing WS_2_ as ETL, the *V*_OC_ in both devices is improved. However, by creating a graded *E*_g_, the *J*_SC_ is improved by the vast spectrum of light that is absorbed. By reducing the thickness of the absorber and ETL in the optimized configuration, we have lowered the *R*_S_ associated with both layers, resulting in an increase in FF. The optimized structure demonstrates an enhanced EQE over a wider wavelength range, as shown in Figure 8d. The graded *E*_g_ structure, which increases light absorption and charge carrier production, especially in spectrum areas where the traditional design is less effective, is responsible for this improvement. Furthermore, the absorber and ETL layers’ optimal thickness reduces optical and electrical losses, guaranteeing effective charge collection and lowering recombination. When these elements work together, incident photons are used more efficiently, which improves device performance overall.

Table 4 summarizes the comparison between the conventional design with uniform bandgap and the optimized graded bandgap. The optimized design outperforms the conventional one in terms of *J*_SC_, FF and efficiency, while a slight decrease in *V*_OC_ is observed (1.08 to 1.03 V). Where the increase in *J*_SC_ (19.07 to 27.66 mA/cm^2^) is mainly attributed to the graded bandgap in the BaZr_1–*x*_Ti_*x*_S_3_ layer, which enhances carrier generation and transport. Consequently, the FF increases from 52.92% to 85.02%, and the efficiency significantly improves from 10.90% to 24.22%, confirming the effectiveness of the proposed optimization strategy.

**Table 4:** Performances of Devices Based on Uniform Bandgap and Optimized Graded Bandgap and the Final Parameters Used for Every Layer

	structure	uniform bandgap	optimized graded bandgap
Parameters	WS_2_		
	χ (eV)	3.95	3.95
	*E*_g_ (eV)	2.1	2.1
	*N*_d_ (cm^–3^)	1 × 10^19^	1 × 10^19^
	*N*_t_ (cm^–3^)	1 × 10^14^	1 × 10^14^
	Thickness (nm)	60	60
	BaZr_1‑*x*_Ti*_x_* S_3_		
	*x*	0.05	0.05 to 0.55
	*E*_g_ (eV)	1.63	1.63 to 1.28
	*N*_t_ (cm^–3^)	1 × 10^15^	1 × 10^13^
	Thickness (nm)	500	300
	Cu_2_O		
	χ (eV)	3.2	3.2
	*E*_g_ (eV)	2.17	2.17
	*N*_a_ (cm^–3^)	1 × 10^18^	1 × 10^18^
	Thickness (nm)	100	100
	*N*_t_ (cm^–2^)	1 × 10^15^	1 × 10^15^
Yields	*V*_oc_ (V)	1.08	1.03
	*J*_SC_ (mA/cm^2^)	19.073	27.66
	*J*_SC_ (mA/cm^2^) *V*_OC_ (V) FF(%)	52.92	85.02
	η (%)	10.90	24.22

## Conclusion

This study emphasizes the importance of optimizing the graded bandgap absorber layer in BaZr_1–*x*_Ti_*x*_S_3_ PSCs to enhance overall performance. It employs a novel simulation-based approach that combines two key strategies: the implementation of a compositionally graded bandgap in BaZr_1-x_Ti_x_S_3_ using a β-function profile, and the integration of WS_2_ as an ETL, resulting in the final device structure FTO/WS_2_/BaZr_1-x_Ti_x_S_3_/Cu_2_O/Au. The proposed design, featuring an optimized β-function graded profile, demonstrates superior performance at an optimized absorber thickness between 200 and 300 nm, achieving a *J*_SC_ of 27.66 mA/cm^2^, an FF of 85.02%, and a PCE of approximately 24.22%. The findings reveal that implementing a well-engineered graded bandgap improves device efficiency by promoting optimal band alignment, which supports efficient charge extraction. This strategy not only enhances light absorption but also reduces recombination losses, leading to a significant increase in solar cell performance. The results highlight the vital role of thoughtful material selection, precise bandgap tuning, and careful layer optimization in advancing PSC efficiency. These insights provide a foundation for the development of advanced next-generation green solar energy technologies.

## Data Availability

All data generated or analyzed during this study are included in this published article. Additional details supporting the findings of this study are available within the manuscript, figures, and tables.
